# Trait-Level Resilience in Pet Dogs—Development of the Lincoln Canine Adaptability Resilience Scale (L-CARS)

**DOI:** 10.3390/ani13050859

**Published:** 2023-02-26

**Authors:** Eilidh L. M. Mackay, Helen Zulch, Daniel S. Mills

**Affiliations:** Department of Life Sciences, University of Lincoln, Lincoln, Lincs LN6 7DL, UK

**Keywords:** behaviour, Lincoln canine adaptability and resilience scale, L-CARS, dog, psychometric scale, resilience, trait

## Abstract

**Simple Summary:**

Dogs are exposed to many stressors and appear to vary in their ability to cope with these, but the concept of resilience (the ability to bounce back after adversity), so important in the human field, has not been specifically studied in this species. This study examined the validity of this concept in dogs, through the development of a reliable instrument to measure resilience as a trait and a subsequent assessment of its psychometric properties. A survey was developed and distributed online to dog owners. Items covered demographic factors and the presence of problem behaviours, as well as 19 items related to three domains of resilience described in the human literature, which might reflect trait-level resilience in dogs. Here, 1084 completed responses were returned, and 329 dog owners completed the survey a second time 6–8 weeks later to assess intra-rater reliability. An analysis of the structure of the resilience items was performed on those found reliable. This revealed a two-component solution, one reflecting “Adaptability/behavioural flexibility” and the other “Perseverance”, which are in line with two aspects of the human conceptualisation of resilience. Good agreement was found with expected correlates, such as problem behaviour, enhancing the validity of this scale for the assessment of resilience in dogs.

**Abstract:**

The concept of psychological resilience is well-explored in the human literature and is often described as the ability to ‘bounce back’ following adversity. However, it remains a neglected research area in dogs despite observations that like humans, dogs vary in their ability to cope with stress. This study aimed to develop the first canine ‘resilience’ scale. An on-line survey was developed for owners. This covered demographics, medical/behavioural history of the dog, and 19 potential resilience items assessed using a 5-point Likert scale; 1084 complete responses were received during the survey period, with 329 respondents subsequently completing the questionnaire a second time, 6–8 weeks later. Intra-rater reliability was assessed, and only reliable items retained. A principal component analysis (PCA) with varimax rotation was then performed with components extracted on the basis of the inspection of scree plots and the Kaiser criterion. Items were retained if they loaded >0.4 onto one of the components but removed if they cross-loaded onto more than one component. This resulted in a 14-item, 2-component solution. One component appeared to describe “Adaptability/behavioural flexibility” and the other “Perseverance”, which are described in the human literature on resilience. Predictive validity was established for expected correlates, such as problem behaviour. The resulting instrument was called the Lincoln Canine Adaptability and Resilience Scale (L-CARS) and is the first to be developed for the assessment of resilience in dogs.

## 1. Introduction

Psychological resilience is generally regarded as the ability to ‘bounce back’ following adversity or trauma [1,2,3,4]. It is frequently discussed in the human literature in terms of exploring the different ways that individuals respond to difficult life events [5,6,7,8]. Anecdotally, dogs show great variation between individuals in their coping abilities; some appear to recover rapidly from what we would perceive as quite significant trauma, whilst others struggle following subjectively milder disturbances. However, whilst of interest and importance [9], differences in resilience in dogs remain an under-researched topic.

In humans, resilience is often considered within the context of the risk of mental health issues and psychiatric disturbance [10,11,12]. Lower resilience scores have been found to correlate with neuroticism [13] (a personality trait indicating tendencies towards negative emotions and emotional instability) and specific disorders, such as post-traumatic stress disorder (PTSD) and generalised anxiety [14]. Those considered less resilient carry an increased risk of suffering from a range of negative psychological states, and it seems reasonable to suppose that the same might occur for dogs. Dogs face a variety of commonplace stressors in their lives, such as during veterinary visits [15,16], car travel [17] and kennelling [18]. Whilst various studies have explored risk factors for behavioural complaints involving negative effects [19,20], the literature lacks a focus on factors, such as resilience, which may protect against the initial development of such problems.

There are numerous validated psychometric resilience scales for use in humans [13,21,22,23,24,25], and this may reflect a lack of consensus regarding the definition [1,3,4,26,27]. Studies have generally described resilience from one or more of three perspectives: (a) as a set of inherent character *traits* (often referred to as ‘trait resilience’); (b) as part of a dynamic *process*, of adapting and recovering from adverse events; or c) as an *outcome*, which evaluates resilience based on the favourability of the outcome itself [11,27,28,29]. The importance of consistent terminology to the development of the scientific basis of clinical animal behaviour has recently been highlighted [30]. Given the complex and clearly multi-faceted nature of psychological resilience as a construct, we chose to focus on ‘trait resilience’ in the current study. This assumes that some individuals carry personal characteristics that increase their ability to cope in the face of adversity and allow positive adaptation to new circumstances [11,13] and was therefore deemed most likely to be measurable between individuals via a psychometric scale.

Despite the inability of non-human animals to self-report, the development of psychometric scales using owners as proxy responders for the evaluation of temperament traits has already been applied successfully to develop valid scales for several other traits [31,32,33]. Therefore, the current study aimed to use a similar approach to that used previously [31,32,33], to develop a psychometric scale for use in dogs to assess trait resilience and determine the evidence of validity.

## 2. Materials and Methods

The questionnaire was created in Qualtrics^®^XM0722 as an on-line questionnaire, written in British English. The questionnaire comprised two sections.

### 2.1. Demographic Data

Demographic data were collected relating to both the owner (country currently living in) and dog (age; breed; sex and neuter status; presence of any medical, behavioural or painful conditions; presence of an unusual gait or movement; whether any prescription medications, supplements/nutraceuticals or pheromone products are used). Some factors were included based on extrapolation from the human literature, such as age and gender [34]. Other factors were included because it was hypothesised that they may impact canine resilience, such as the impact of pain on behaviour [35].

### 2.2. Resilience and Related Items

An extensive review of the human resilience literature was performed to define trait resilience in a way that would be relevant to pet dogs (face validity). Our definition was based on the three domains described by Maltby and colleagues [27]:

1. *Engineering resilience*—an individual’s ability to return to normal (‘bounce back’) following adversity;
2. *Ecological resilience*—the capacity to tolerate/withstand disruptions, with the ability to maintain composure during challenges (focus on ‘best efforts’, perseverance);
3. *Adaptive capacity*—the ability to handle and adapt to change, i.e., flexibility in strategies and even enjoyment of change.

These domains were then used to create statements (potential items in the instrument) that might reflect the equivalent state as a form of trait resilience in dogs, which could be answered by a typical owner. For example, for the *Engineering resilience* domain there was the item: *“My dog takes a long time to recover from set-backs in life…”*; for the *Ecological resilience* domain, there was the item: *“My dog will persevere even when they do not succeed in something straight away...”* (to reflect ‘best efforts’ when faced with challenges); and for the *Adaptive capacity* domain, there was the item: *“I would regard my dog to be very adaptable* i.e., *able to fit into any situation”*. An item was also incorporated to allow assessments of convergent validity with the construct of interest: *“I believe my dog to be a resilient individual* i.e., *able to cope with, ‘bounce back’ from and/or adapt to adversity or change”*. Nineteen items were included in our preliminary questionnaire, which included 6 items for each of the three described domains and 1 item for the assessment of convergent validity [see Supplementary Material S1 for a full list of initial items]. A 5-point Likert scale was used to rate each of these items, ranging from “Strongly Agree” through “Mainly Agree”, “Partly Agree, Partly Disagree”, “Mainly Disagree” to “Strongly Disagree”, with six items reverse scored to reduce the risk of response set acquiescence [36]; an “N/A or Don’t Know” option was also included to avoid participants feeling forced to select a response. Similar rating systems have been used in other validated canine temperament scales [31,32,33]. A random order generator function was used on Qualtrics^®^XM to minimise the risk of grouping items in relation to one of the three facets [27], which might result in an order bias.

Finally, questions pertaining to the presence or absence of a range of commonly reported behaviour problems were included given the correlations between resilience and psychiatric disorders described in the human literature [14].

### 2.3. Questionnaire Piloting and Distribution

The questionnaire was trialled with a pilot sample of 5 individual dog owners to assess comprehensibility and clarity. Minor amendments were made, and the questionnaire was subsequently made live to the public, under the heading: *“Can resilience be measured as a trait in pet dogs?”* [see Supplementary Material S2, for full questionnaire].

An advertisement containing an electronic link to the questionnaire was created. This was distributed via several social media platforms (Facebook, Instagram and Twitter), as well as via direct contacts. Both methods had the potential for snowballing sampling effects.

The inclusion criteria required participants to be a minimum of 18 years of age and be able to provide informed consent. It was also specified that they *currently* owned the dog for whom they intended to complete the questionnaire. Participants owning multiple dogs were asked to complete it for only one dog and were requested to select the dog they had owned longest. Participants were requested to read the participant information sheet and complete an electronic consent form before they could take part in the study. The original questionnaire was made available from the 4th until 29th of July 2022. Participants were given the option to provide an email address to enable future intra-rater reliability testing.

Respondents who provided an email address to the original circulation were contacted again a minimum of 6 weeks (maximum 7 weeks) after they had completed the first questionnaire. They were asked to complete the questionnaire again for the same dog so that the intra-rater reliability of the items could be assessed. Any questionnaires completed within 1 week from being contacted were included in our analysis, giving a minimum 6-week and maximum 8-week window between first and second questionnaires. The questionnaire items and layout were unchanged between surveys.

### 2.4. Statistical Analysis

Statistical analyses were conducted using R 4.2.1.

#### 2.4.1. Intra-Rater Reliability Assessment

Percentage agreements were calculated for each item, i.e., how many respondents selected the same response between the first and second completion of the questionnaire. Following this, non-parametric Spearman’s rank order correlations and Wilcoxon signed rank tests were performed on each of the items in turn, with any *“N/A or Don’t Know”* responses excluded from this part of the analysis. Items were excluded on the basis of weak correlations (Spearman’s rank order correlation <0.4) or significant differences (*p* < 0.05) between the first and second questionnaire (Wilcoxon signed rank test). Bonferroni adjustments were applied to *p*-values to correct for multiple testing.

#### 2.4.2. Psychometric Evaluation

Items were scored to reflect the hypothesis that higher scoring individuals would have higher resilience. The Likert scale responses were converted into numerical scores: typically, 5 for “Strongly Agree”; 4 for “Mainly Agree”; 3 for “Partly Agree, Partly Disagree; 2 for “Mainly Disagree” and 1 for “Strongly Disagree”, but the negatively worded items were reverse scored [(**R**) in Supplementary Material S1] (i.e., “Strongly Agree” becomes 1 and “Strongly Disagree” becomes 5).

Given the response rate and potential complications that can occur when imputing missing data [37], it was decided that records would be deleted for respondents who answered 3 or more of the 19 resilience items with an *“N/A or Don’t Know”* response. The remaining data were then sorted into complete and incomplete datasets for a subsequent comparison of item scores between these two populations, using Wilcoxon rank sum tests. Bonferroni adjustments were applied for multiple testing. 

Using the complete datasets (i.e., any individual response sets with no *“N/A or Don’t Know”* responses), a between-items correlation matrix was created for the resilience items. Any items suggesting singularity by lacking any correlations >0.2 with any other item or suggesting multicollinearity and redundancy due to correlations >0.8 with another item were removed [32].

A principal component analysis (PCA) with varimax rotation was then performed on the remaining items [38]. The aim of a PCA is to determine whether a large number of interrelated variables can be reduced into a smaller number of measures [39]. A varimax rotation was selected to maximise the difference between factors [40]. The number of components to be extracted was decided using the point of inflection on the scree plot [41] along with the Kaiser Criterion, which proposes the retention of components with an Eigenvalue >1 [42]. For interpretative purposes, items were deemed to load onto a given component if the loading was >0.4 [39]. Items that did not load >0.4 onto any component or those that cross-loaded (i.e., loaded >0.4 onto more than one component) were removed from further analysis.

The internal consistency of the remaining scale was measured using Cronbach’s alpha [33]. Relationships between the principal component scores and demographic data were explored using a multifactor linear model, with each principal component score as the dependent variable in turn. The independent variables were fitted into the same model and included the fixed factors country, age (presented in range categories), breed, sex, neuter status, presence of a medical condition, whether the dog takes any prescription medications, whether any supplements or nutraceuticals are taken, whether any pheromone products are used and finally the presence/absence of the 7 named problem behaviours asked in the original questionnaire (see Q10 in Supplementary Material S2). Where significant differences were found, post-hoc tests were performed using Bonferroni adjustments.

In order to establish normal ranges for the instrument, scores were calculated for individuals for each of the principal components (PCs), using the combination of complete and incomplete datasets. These were calculated by dividing the sum of the scores for the items in the given component by the number of items scored multiplied by 5 (since 5 is the maximum score). Items answered *“N/A or Don’t Know”* were excluded from the calculation, and so the related PC was divided by a smaller number. This produced a component score with a possible range of 0.2–1.0 which allowed for standardisation of the scores and a comparison between dogs. A ‘normal range’ for each principal component was then calculated based on individuals remaining after the removal of any factors found in the preceding linear models to significantly affect the PC score.

## 3. Results

### 3.1. Responses

Here, 1441 responses were collected; 248 were excluded for being incomplete. The rate of *“N/A or Don’t Know”* responses was generally low with the highest rate on Item 1 at 12.6%; 82 responses contained ≥3 *“N/A or Don’t Know”* answers to the potential resilience items, and so were deleted from the dataset. Missing potential resilience data remained in only 27 responses (2.3%). Missing rates were very low, with the highest rate on Item 7 at 0.5%. This left 1084 complete response sets. No evidence of response sets was identified among respondents.

### 3.2. Demographics

Most respondents were from the United Kingdom (*n* = 659, 60.8%), followed by the USA (*n* = 159, 14.7%). Respondents from other countries were classified as “Other countries” for the purpose of analysis. This included 76 (7.0%) from Australia, 36 (3.3%) from Canada, 12 (1.1%) from Ireland, 3 (0.3%) from New Zealand, and 1 (0.1%) from Jersey; 77 respondents (7.1%) selected the “Other/Not on list” option, and 61 respondents (5.6%) did not provide a country.

Whilst only a small number of dogs (*n* = 5, 0.5%) was observed in the *“6 months old or less”* category, data spanned all five age categories used in the questionnaire (see Supplementary Material S3).

The majority of dogs (*n* = 667, 61.5%) were reported to be pure bred, with 109 breeds represented in the data. A total of 322 (29.7%) reported their dog to be a “Cross-breed” (defined as *“a mix between two or more breeds”*). Eleven breeds, which each accounted for >1% of the study population, were classified as individual breeds for the purpose of the analysis. All specific breeds comprising <1% were labelled “Other pure-breed” (*n* = 98) (see Supplementary Material S3 for details). A small number (*n* = 72, 6.6%) did not provide breed details.

There was a relatively even sex split (male = 572, female = 512) with the majority of dogs reported to be neutered: male neutered (*n* = 427, 39.4%), female neutered (*n* = 422, 38.9%), male entire (*n* = 145, 13.4%) and female entire (*n* = 90, 8.3%).

The percentage of respondents who reported their dog as having a pre-existing medical condition was 28.9% (*n* = 313), and 68.4% (*n* = 214) of these reported that the condition(s) caused pain. A total of 391 respondents (36.1%) reported an unusual gait or movement, and over one quarter of respondents (*n* = 309, 28.5%) reported their dog to be currently taking a prescription medication.

Nearly three quarters of respondents (*n* = 785, 72.4%) reported their dog to have at least one problem behaviour. Table 1 details the prevalence of individual problem behaviours within our sample.

### 3.3. Intra-Rater Reliability (See Supplementary Material S4 for Full Data)

Complete responses for the second questionnaire totalled 329. Of these, three provided an email address that could not be matched to the one in the original questionnaire, and nine did not provide an email address. This left 317 completed paired questionnaires available for this part of the analysis. All items other than item 5 and 13 displayed a percentage agreement greater than 40%, with the majority greater than 50%.

Spearman’s rank order correlations performed on each of the items revealed strong correlations (rho = 0.5–1.0) for 18 of the 19 items, with one item (Item 2) displaying a moderate correlation in the upper part of the range (rho = 0.3–0.49). Wilcoxon signed rank tests revealed significant differences in median test–retest scores for three items: Item 1, Item 3 and Item 13—these items were therefore removed from the questionnaire.

### 3.4. Inter-Item Correlations and Principal Component Analysis (PCA) (See Supplementary Material S5 for Supporting Details)

The correlation matrix of the remaining 16 items revealed that all items satisfied the requirements of correlations >0.4 with at least one other item whilst having no correlations >0.8 with another item.

A PCA with Varimax orthogonal rotation was performed on the remaining 16 reliable items, following a Kaiser–Meyer–Olkin (KMO) Measure of Sampling Adequacy [43] and Bartlett’s Test of Sphericity [44] indicating the suitability for this type of analysis [KMO = 0.93; Bartlett’s Test of Sphericity was highly significant χ^2^ (120) = 6259.14 (*p* < 0.001)].

The point of inflection on the scree plot [41] suggested a possible two or three component solution; however, the Kaiser criterion [42] supported the extraction of only two components. These two components explained 55.2% of the variance.

Item 2 did not load >0.4 onto either component and was removed from subsequent analysis. Item 16 cross-loaded onto both components, so it was also removed. The remaining 14 items displayed sufficient loading (>0.4) onto only one of the components and so were retained.

### 3.5. Comparison of ‘Complete’ and ‘Incomplete’ Datasets

There were no significant differences identified between medians in the complete and incomplete datasets for either of the principal components (PC1 and PC2) or for any of the remaining resilience items (Bonferroni-adjusted *p* > 0.05, see Supplementary Materials S6 for details).

### 3.6. Construct Validity of Components

The remaining two components with their 14 items were assessed for face validity in relation to relevant resilience constructs. PC1 (11 items) appeared to reflect Adaptability/Behavioural Flexibility and PC2 (3 items), Perseverance (Table 2). The main construct item (Item 19: I believe my dog to be a resilient individual i.e., …) loaded strongly (0.79) onto principal component 1 (PC1). The resulting instrument was called the Lincoln Canine Adaptability and Resilience Scale (L-CARS)—See Supplementary Material S7 for a copy of the final instrument.

### 3.7. Internal Consistency

Overall Cronbach’s alpha for the 14-item scale was = 0.896. Whilst the deletion of PC2 resulted in a very minor increase to the Cronbach’s alpha score (=0.907, see Supplementary Materials S8), this was considered trivial, and so it was elected to retain both components in the Canine Adaptability and Resilience Scale.

### 3.8. Relationship between Principal Component Scores and Demographics

The results of the multifactor linear models exploring the effect of demographic variables on the principal component scores (Adaptability/Behavioural Flexibility and Perseverance scores) are summarised below and in Table 3 (details of the content of the variables are included in Supplementary Materials S8).

There was a significant relationship with the use of pheromone products on the Adaptability/Behavioural Flexibility Score with the use of pheromone products (diffuser, spray and/or collar) related to a lower PC1 Score (Linear Model: F_1,889_ = 5.660, Bonferroni-adjusted *p* = 0.035). There were also significant effects relating to the Adaptability/Behavioural Flexibility Score and the following behaviour problems identified by the owner: unfriendly or aggressive behaviour towards dogs (Linear Model: F_1,889_ = 75.109, Bonferroni-adjusted *p* < 0.001); fears or phobias, e.g., noise reactivity/fear, fear of car travel (Linear Model: F_1,889_ = 243.870, Bonferroni-adjusted *p* < 0.001); separation-related behaviour problem (Linear Model: F_1,889_ = 31.687, Bonferroni-adjusted *p* < 0.001); unfriendly or aggressive behaviour towards people (Linear Model: F_1,889_ = 59.557, Bonferroni-adjusted *p* < 0.001); other problem behaviour not listed (Linear Model: F_1,889_ = 32.975, Bonferroni-adjusted *p* < 0.001). In each case the PC Score was significantly lower when the behaviour problem was present. There were no other significant relationships with this PC.

**Figure 1:**
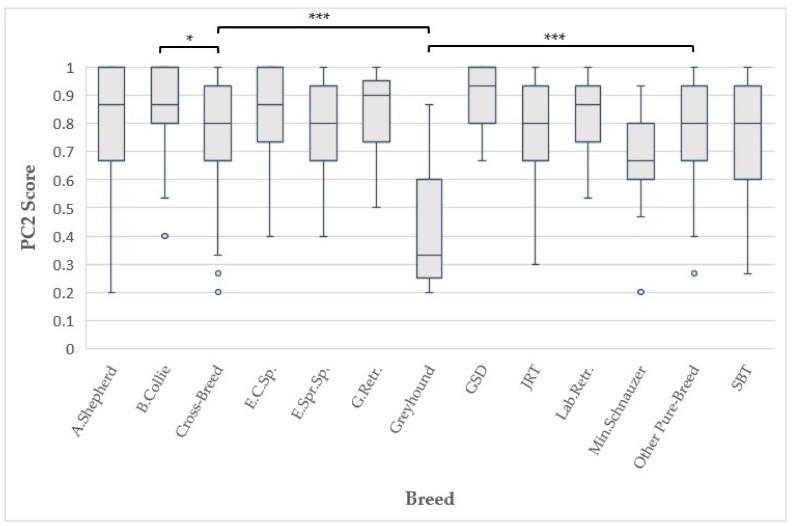
Relationship between breed and Perseverance Score (PC2) (significance codes: * = *p* < 0.05, *** = *p*< 0.001). **Key:** A. Shepherd = Australian Shepherd; B. Collie = Border Collie; E.C.Sp. = English Cocker Spaniel; E.Spr.Sp. = English Springer Spaniel; G.Retr. = Golden Retriever; GSD = German Shepherd Dog; JRT = Jack Russell Terrier; Lab.Retr. = Labrador Retriever; Min.Schnauzer = Miniature Schnauzer; SBT = Staffordshire Bull Terrier.

There was a significant effect of breed on the Perseverance score (Linear Model: F_12,889_ = 8.269, Bonferroni-adjusted *p* < 0.001 Figure 1). Post-hoc analyses compared firstly all pure-breed to cross-breed dogs and then the 11 most common breeds with both “Other pure-breed” and “Cross-breed” dogs. Border Collies were found to have a significantly higher Perseverance score than cross-breeds (Linear Model: F_1,889_ = 10.083, Bonferroni-adjusted *p* = 0.037); Greyhounds were found to have a significantly lower Perseverance score than both ‘other pure-breeds’ (Linear Model: F_1,889_ = 51.941, Bonferroni-adjusted *p* < 0.001) and cross-breed dogs (Linear Model: F_1,889_ = 54.085, Bonferroni-adjusted *p* < 0.001). No significant differences were detected between pure-breeds and cross-breeds overall. Perseverance scores were also significantly lower for dogs with fears and phobias. All other comparisons were not statistically significant.

### 3.9. Principal Component Scores

Principal component scores were calculated for each individual dog based on the remaining 14 items. This was performed on the entire dataset (*n* = 1084) as described in the statistical methods. The scores were found to range from 0.2 to 1.0 (see Supplementary Material S8 for full descriptive statistics). The ‘normal ranges’ for dogs for Adaptability/Behavioural Flexibility (PC1) and Perseverance (PC2) within L-CARS were calculated for individuals following the removal of those with non-permanent characteristics identified as resulting in a significant difference in the PC score. In other words, the Adaptability/Behavioural Flexibility ‘normal range’ was calculated from scores of individuals not using pheromones and who did not have any of the following problem behaviours: aggressive to dogs, fears or phobias, separation-related behaviour problems, aggressive to people, or another un-named problem behaviours. This gave a mean value ± standard deviation of 0.82 ± 0.10. The Perseverance ‘normal range’ was calculated based on all individuals other than those with reported fears or phobias and produced a normal reference range of 0.81 ± 0.17.

Finally, ranges for each PC were calculated for each of the problem behaviours: aggressive to dogs (PC1 = 0.61 ± 0.15; PC2 = 0.79 ± 0.18); fears or phobias (PC1 = 0.59 ± 0.15; PC2 = 0.76 ± 0.19); separation related behaviour problem (PC1 = 0.63 ± 0.15; PC2 = 0.77 ± 0.19); hyperactivity/overexcitement (PC1 = 0.65 ± 0.16; PC2 = 0.82 ± 0.16); aggressive to people (PC1 = 0.56 ± 0.15; PC2 = 0.77 ± 0.18), repetitive behaviour (PC1 = 0.65 ± 0.17; PC2 = 0.75 ± 0.20); and unnamed problem behaviour (PC1 = 0.59 ± 0.18; PC2 = 0.77 ± 0.20).

## 4. Discussion

We successfully developed a robust and concise psychometric instrument “The Lincoln Canine Adaptability Resilience Scale (L-CARS)”, which provides a foundation for the future assessment of trait-level resilience in pet dogs.

Conciseness is important to the practicality of the instrument. A scale that is too long is less likely to be used; however, removing too many items can come at a cost through the elimination of potentially meaningful items. A balance must also be struck between the retention and deletion of items in order to maximise specificity, whilst retaining sensitivity. We elected to remove the items that cross-loaded to increase the specificity of the constituent components. Furthermore, as these items did not relate to specific scenarios, their removal was unlikely to greatly reduce sensitivity. PC1 appeared to reflect “Adaptability/behavioural flexibility”, and the constituent items seem to align with both the “Engineering Resilience” (ability to bounce back) and the “Adaptive Capacity” facets described by Maltby et al. [27], and this perhaps reflects a common understanding of resilience. It is therefore not surprising that the construct item specifically asking about resilience loaded strongly on this PC. A dog scoring highly in this domain may be expected to adapt well (and possibly even flourish) in response to change and be able to adjust their behaviour in accordance with differing contexts (‘Behavioural Flexibility’). By contrast, PC2 appeared to reflect “Perseverance”, which perhaps aligns most closely with the concept of “Ecological Resilience” described by these authors [27]. In humans, this relates to maintaining best efforts and composure in the face of adversity, but in non-human animals may be recognised as an individual persisting in the face of disruption, which reflects the content of the items of the second PC. This is perhaps a less commonly appreciated aspect of resilience, and so the poor loading with the main construct item is not surprising. It is also interesting to note that the perseverance component was associated more with breed-related differences than problem behaviour, and so this might reflect a more general behavioural (rather than psychological) trait. Nonetheless, the two-component solution described here appears to cover the conceptual breadth of resilience described in humans [27], and so the scale has good face validity.

Concurrent validity concerns the degree to which a measure agrees with an established indicator of the facet of interest assessed at the same time. In this regard, the occurrence of behaviour problems associated with fear and anxiety in dogs recorded within the survey may be of particular interest, given the associations identified between resilience and related issues in humans [13,14]. It is notable that problems, such as fears/phobias, aggressive behaviour and separation-related issues, were strongly related to lower “Adaptability/Behavioural Flexibility” scores, while other problems, such as hyperactivity (which has been found to be unrelated to negative stress levels [45]) and repetitive behaviour problems (which are commonly related to frustration [46] or play in dogs, e.g., [47]) were not. This reinforces the suggestion that this component is the main measure of resilience within L-CARS. However, the relationship between the “Perseverance” component and fears/phobias indicates that this second component may also be of importance, hence our decision to retain it within the final instrument. Future work should explore the relationship between these components and other measures of relevance to resilience, including physiological correlates [48], and further evaluate its discriminant validity.

Both principal component scores displayed negatively skewed distributions with a ‘normal’ mean value around 0.8 out of a possible maximum score of 1.0 and a standard deviation of around 0.1. Although it is possible that these skews arise from a recruitment bias resulting in the attraction of enthusiastic, knowledgeable dog owners who are proud of their dog’s purported ‘resilience’, the prevalence of problem behaviour in our population would suggest that this is not the case.

We accept that this instrument assesses owner perception of their dogs’ behaviour and it is possible that owners overestimate their dogs’ resilience. The literature shows that without training, people can be poor interpreters of dog behaviour [49]. For this reason, further investigation into the validity of this instrument needs to be undertaken. This should include the testing of inter-rater reliability, as well as an examination of convergent validity by investigating physiological and independent behavioural correlates, as has been done in the development of other psychometric scales [50,51].

However, even if future work confirms a skew within the dog population, this is unlikely to be of great importance given the most probable application of the scale will be in relation to predictions based on those who are less resilient, rather than differentiation between ‘normal’ and exceptionally resilient individuals. In particular, the relationship between lower scores and the risk of developing a range of behaviour and welfare conditions of concern may be of particular value.

At present, the relationships identified with problem behaviour are correlational and the direction of any causal association cannot be identified. It might be that animals with the problems described are seen to be less resilient or that less resilient individuals are at greater risk. In their regard, the association with the use of pheromone-related products is of interest. Whilst the same caveats regarding the causal nature of the relationship apply, it is worth noting that these products were developed in order to help individuals cope with a range of stressors [52]. This scale may therefore provide a useful instrument for evaluating and monitoring the more general effects of these products and other measures aimed at increasing resilience.

Although gender and age differences in resilience are widely reported in humans [53,54,55,56,57], our data suggest no relationship between resilience and gender, neuter status or age. The reasons for this deserve further investigation. There was also an absence of significant effects in relation to the presence of medical conditions, owner-perceived presence of pain or owner-perceived presence of an abnormal gait, despite the known impact these factors can have on behaviour in dogs [35]. However, these findings are based on an owner-report, which is a common potential limitation of survey-based research. In this regard, it is worth noting that many medical conditions, such as osteoarthritis, are often unrecognised by dog owners [58,59], and some inherited disorders may be regarded as ‘normal’ for the breed [60], and so there may be many false negatives within the unaffected group. Furthermore, it is interesting that the number reporting an abnormal gait was higher than the number reporting a medical condition, supporting the notion that medical conditions may be under-reported here. Further work associated with more specific diagnostics is warranted to explore this further.

## 5. Conclusions

Understanding the factors that promote a dog’s ability to cope with adversity is important for our ability to promote positive welfare. Having a means of assessing resilience is a first step to future studies into this area. Overall, L-CARS showed good correlation with many expected resilience-related associations, and there were no significant relationships that could not be explained in relation to resilience (i.e., no evidence to falsify the validity of the scale). Although it is important that investigation is undertaken to further validate the scale, we believe that L-CARS has good construct validity and provides a useful foundation for future research into canine resilience.

## Figures and Tables

**Table 1:** Prevalence of problem behaviours within the sample.

Problem Behaviour Category	Number	Percentage (%)
“Unfriendly or aggressive behaviour towards dogs”	407	37.5
“Fears or phobias e.g., noise reactivity/fear, fear of car travel”	355	32.7
“Separation related behaviour problem”	237	21.9
“General hyperactivity/overexcitement”	233	20.6
“Unfriendly or aggressive behaviour towards people”	188	17.3
“Repetitive behaviour including self-mutilation (e.g., compulsive licking/chewing), tail-chasing or shadow chasing”	108	10.0
“Other problem behaviour not listed”	96	8.9

**Table 2:** Final 14-item Lincoln Canine Adaptability and Resilience Scale (L-CARS), 2-component solution based on PCA with varimax rotation, with biological interpretations and variance explained by each component.

PC	Item Number	Item (R) = Reverse Scored	Variance Explained (%)
PC1 Adaptability/ behavioural flexibility	4	If something frightened my dog, he/she would be nervous to return to that location for a long time afterwards (R)	42.61
	5	If my dog were to have a bad experience with another individual (dog or person), he/she would forget about it quickly and not hold onto it	
	6	If something were to startle or frighten my dog, he/she would remain on edge for some time afterwards (R)	
	8	My dog does not get upset easily	
	9	My dog generally takes stressful situations in their stride	
	11	My dog will sometimes seem out of sorts for no apparent reason (R)	
	12	If another dog has a negative reaction to something, my dog is likely to become upset too (R)	
	14	My dog enjoys anything that is new or unusual, e.g., objects, animals or anything they have not seen before	
	15	I would regard my dog to be very adaptable, i.e., able to fit into any situation	
	18	If something unexpected were to happen and my life circumstances were to change, I know my dog could cope	
	19	I believe my dog to be a resilient individual, i.e., able to cope with, ‘bounce back’ from and/or adapt to, adversity or change	
PC2 Perseverance	7	My dog always tries his/her hardest even when the task is difficult	12.61
	10	My dog will persevere even when they do not succeed in something straight away, e.g., when learning a new trick, when trying to solve a puzzle, etc.	
	17	My dog enjoys challenges, e.g., learning new tricks, finding hidden items, solving a difficult task	

**Table 3:** Details of outputs from linear models using the demographic variables listed, with PC1 and PC2 as the dependent variable (significance codes: * = *p* < 0.05, *** = *p* < 0.001).

	PC1 Adaptability/Behavioural Flexibility			PC2 Perseverance		
Demographic Variable	df	*F*	*p*	df	*F*	*p*
Country	2	3.179	0.084	2	1.338	0.526
Age	4	1.645	0.322	4	2.083	0.162
Breed	12	1.605	0.170	12	8.269	<0.001 ***
Sex	1	0.263	1.000	1	0.918	0.677
Neuter Status	1	1.819	0.356	1	1.290	0.513
Pre-existing medical condition	1	1.595	0.414	1	0.298	1.000
Takes prescription medication	1	2.931	0.175	1	2.912	0.177
Takes supplements or nutraceuticals	1	0.312	1.000	1	1.828	0.353
Uses pheromone products	1	5.660	0.035 *	1	0.087	1.000
Problem Behaviour Category:						
• Aggressive to dogs	1	75.109	<0.001 ***	1	0.030	1.000
• Fears/phobias	1	243.870	<0.001 ***	1	9.749	0.026 *
• Separation related behaviour problem	1	31.687	<0.001 ***	1	3.071	1.000
• General hyperactivity/overexcitement	1	1.995	1.000	1	5.037	0.350
• Aggressive to people	1	59.557	<0.001 ***	1	3.156	1.000
• Repetitive behaviour	1	2.930	1.000	1	8.366	0.055
• Other problem behaviour	1	32.975	<0.001 ***	1	3.102	1.000

## Data Availability

The data that support the findings of this study are available from the corresponding authors upon reasonable request.

## Supplementary Materials

The following supporting information can be downloaded at: https://www.mdpi.com/article/10.3390/ani13050859/s1, S1: Preliminary ‘resilience’ items used in Likert Scale and Scoring system; S2: Participant information sheet and full questionnaire; S3: Basic demographic information relating to initial survey; S4: Initial metrics on item quality; S5: Inter-item correlations and principal component analysis (PCA); S6: Metrics relating to comparison of ‘complete’ and ‘incomplete’ datasets for The Lincoln Canine Adaptability and Resilience Scale (L-CARS); S7: The Lincoln Canine Adaptability Resilience Scale (L-CARS); S8: Metrics relating to final questionnaire: The Lincoln Canine Adaptability and Resilience Scale (L-CARS).
