# Supplementary material for: Trait-Level Resilience in Pet Dogs—Development of the Lincoln Canine Adaptability Resilience Scale (L-CARS)
Source: Animals (Basel). 2023 Feb 26;13(5):859. doi: 10.3390/ani13050859 (PMC10000169; doi:10.3390/ani13050859)
Supplement: Supplementary file 1 [file animals-13-00859-s001.zip › animals-2221003-supplementary.pdf]

animals

Article

Trait-Level Resilience in Pet Dogs—Development of the Lincoln Canine Adaptability Resilience Scale (L-CARS)

Eilidh L.M. Mackay, Helen Zulch * and Daniel S. Mills *

Dept. of Life Sciences, University of Lincoln, Lincoln, Lincs LN6 7DL, UK
* Correspondence: hzulch@lincoln.ac.uk (H.Z.); dmills@lincoln.ac.uk (D.S.M.)

S1: Preliminary 'resilience' items used in Likert Scale and Scoring system

(R) denotes items for reverse scored items

Numbers in parentheses after each statement refer to which of the three domains (listed below) described by Maltby et al. inspired their development.

1. Engineering resilience – an individual's ability to return to normal ('bounce back') following adversity
2. Ecological resilience - the capacity to tolerate/withstand disruptions, with ability to maintain composure during challenges (focus on 'best efforts', perseverance)
3. Adaptive capacity – the ability to handle and adapt to change i.e. flexibility in strategies and even enjoyment of change

Maltby, J.; Day, L.; Hall, S. Refining Trait Resilience: Identifying Engineering, Ecological, and Adaptive Facets from Extant Measures of Resilience. *PLoS One* **2015**, *10*.

1. My dog takes a long time to recover from set-backs in life e.g. loss of a companion, knock in confidence **(R)** (1)
2. My dog bounces back quickly from illness or injury (1)
3. If something upsets my dog, they tend to get over it quickly (1)
4. If something frightened my dog, he/she would be nervous to return to that location for a long time afterwards **(R)** (1)
5. If my dog were to have a bad experience with another individual (dog or person), he/she would forget about it quickly and not hold onto it (1)
6. If something were to startle or frighten my dog, he/she would remain on edge for some time afterwards **(R)** (1)
7. My dog always tries his/her hardest even when the task is difficult (2)
8. My dog does not get upset easily (2)
9. My dog generally takes stressful situations in their stride (2)
10. My dog will persevere even when they do not succeed in something straight away e.g. when learning a new trick, when trying to solve a puzzle etc. (2)
11. My dog will sometimes seem out of sorts for no apparent reason **(R)** (2)

12. If another dog has a negative reaction to something, my dog is likely to become upset too **(R)** (2)

13. My dog does not cope well with change e.g. changes in work hours, house move **(R)** (3)

14. My dog enjoys anything that is new or unusual e.g. objects, animals or anything they have not seen before (3)
15. I would regard my dog to be very adaptable i.e. able to fit into any situation (3)
16. I would regard my dog to be curious/open to new experiences e.g. enjoys exploring new environments (3)
17. My dog enjoys challenges e.g. learning new tricks, finding hidden items, solving a difficult task (3)
18. If something unexpected were to happen and my life circumstances were to change, I know my dog could cope (3)
19. I believe my dog to be a resilient individual i.e. able to cope with, 'bounce back' from and/or adapt to adversity or change (Item being used to assess convergent validity with the construct of interest)

S2: Participant Information sheet and Full Questionnaire

Participant Information Sheet/Information about the research

Title of Study: Can resilience be measured as a trait in pet dogs?

We are inviting you to take part in a research study. Before you decide, it is important that you know why we are doing the study and what is involved. You will need to read the following information sheet and complete a consent form before you are able to access the questionnaire. Please read the following information carefully.

What is the purpose of the study?

Why is it that some dogs seem to sail through life, irrespective of life's challenges, whilst others struggle with even minor difficulties? In human psychology, the concept of resilience has been used to explain how people respond when faced with trauma or adversity. Resilience, which can be defined as the ability to resist, 'bounce back' and even thrive in the face of adversity may be an important attribute in pet dogs, who can face a variety of stressors in day-to-day life.

There are a number of psychometric 'resilience scales' that are used in humans. However, so far, there is nothing available of this kind in dogs. This research aims to explore whether resilience is a trait that can be measured in pet dogs, and if we find that we can measure it, the study may form the beginning of the development of a psychometric canine resilience scale. Such a scale could help us in many ways including in being able to predict how individual dogs might respond to certain life events and therefore how we can best support their welfare.

This research involves an online questionnaire that will ask you to score your dog based on various aspects of their personality and temperament, and the way in which they respond to a range of scenarios.

Am I eligible to take part?

You are eligible to take part if you are:

- 18 years of age or over
- Able to give informed consent to take part in the questionnaire
- Currently own a pet dog (of any age and type)

NB. This questionnaire must be completed for a dog you **currently own**, not one that you have owned in the past. It must be completed for **only one dog**; we ask you to complete it for the dog you have **owned the longest**.

Do I have to take part?

Participation is completely voluntary. You should only take part if you want to and choosing not to take part will not disadvantage you in any way.

What will I be asked to do?

Before starting the questionnaire, you will have read through this information section. You will then be asked to tick an informed eConsent form, if you wish to proceed.

Should you decide to take part, you will be directed to the online questionnaire which will take around 10-15 minutes to complete. Please ensure you complete this for a dog that you currently own. **If you have multiple dogs, please just complete it for one of them - we ask you to complete it for the one you have owned the longest.**

The questionnaire will be comprised of two sections:

1. Demographic information about you and your dog (country you live in; details about your dog's age, breed, sex and neuter status; presence of any medical, behavioural or painful conditions; whether any medication, supplements or other products are used)
2. Questionnaire items – profiling how your dog copes with stress

At the end, we will give you the option to provide an email address should you be happy to complete the questionnaire again in the future. This is to allow us to assess the reliability of the scale over time so it would need to be completed for the **same dog** a second time. This is entirely voluntary.

Will I be paid expenses for taking part?

You will not be paid to participate in the study.

What are the possible benefits / risks of taking part?

There are very few risks of participating in this study, but there are a few things that you should be aware of. Completing the questionnaire will require approximately 10-15 minutes of your time. You can choose not to finish the survey at any point.

As stated above, you will be given the option to provide an email address should you be happy to complete the questionnaire for a second time. This is entirely optional, but it is important that if you do choose to do this, that you are aware that you are consenting to providing us with personal information. You can change your mind i.e. you can later decide that you no longer want to complete the questionnaire for a second time; there is no obligation. Should you provide an email address you can request removal of your data from the data set up until data has been combined as at that point all data will be

anonymous. You can still request that data linked to your email address be removed from any follow up research.

If you choose not to provide an email address, your data will be supplied anonymously. This does however mean that once you submit your response, you will not be able to withdraw from the study as we will have no way of identifying you.

Will anyone know I have taken part?

For all anonymous data, no one will know you have taken part.

For those providing personal information:

The University of Lincoln (UoL) is the sponsor for this study based in the United Kingdom. We will be using information from you only as required in order to undertake the necessary components of the study. We will act as the data controller for this study. This means that we are responsible for looking after your information and using it properly.

No one else will know you have taken part. The research team will keep all data confidential and secure at all times. The information will be used for no other purposes than that has already been specified. It will be deleted when it is no longer required. There will be no other personal data or sensitive information collected. You will not be able to be identified in any reports or publications.

Where will my data be stored?

The data obtained from the study will be stored securely on the university OneDrive with password protected access. Only the researchers will have access to it. No paper copies will be collected. The data from this study may be put in an Open Access repository for other researchers to use in future research. If so, responses will be anonymised and any personal data (e.g. contact details) will be removed.

We will keep identifiable information (i.e. email address, if provided) until the study is complete. After this point they will be deleted. If you wish to withdraw your email address at any point before this, you can contact us and we will remove it immediately.

What will happen if I don't want to carry on with the study?

If you have completed the study anonymously it will not be possible to remove the data provided, as I will not be able to identify you in any way.

If you provide an email address, you are free to withdraw from this study at any point until data has been combined for analysis; at this point all data is anonymous. You do not need to give a reason for withdrawing from the study and can do so by contacting one of the researchers (contact details provided at end).

If you choose to withdraw from the study, the information you have given us up to this point will be deleted/destroyed. However, once the anonymised data set has been created it will not be possible to remove your anonymised data from the analysis.

What will happen to the results of the research study?

The results of this research will be written up in my thesis for my MSc in Clinical Animal Behaviour from the University of Lincoln, U.K., due on the 26/08/2022. They may also be published in a scientific journal at a later date and / or presented at a scientific conference.

Who is organising and funding the research?

This research is being organised by Dr. Eilidh Mackay and supervised by Dr. Helen Zulch and Professor Daniel Mills at the University of Lincoln.

Who has reviewed the study?

All research conducted by the University of Lincoln is looked at by an independent group of people, called a Research Ethics Committee, to protect your rights, dignity and wellbeing. This study has been reviewed and given favourable opinion by a University of Lincoln Research Ethics Committee [UoL2022_1069].

What if there is a problem?

It is very unlikely that this study would cause you any harm. If you have a concern or a complaint about any aspect of this study, you should ask to speak to the researchers who will do their best to answer your questions. The researchers contact details are given at the end of this information sheet. If you remain unhappy and wish to complain formally, you can make a formal complaint through the University complaints procedure or by contacting ethics@lincoln.ac.uk.

Further information and contact details

Contact details

Lead researcher:

Dr. Eilidh Mackay – 25637250@students.lincoln.ac.uk

Supervisors:

Dr. Helen Zulch – hzulch@lincoln.ac.uk

Professor Daniel Mills – dmills@lincoln.ac.uk

Information compliance

The University of Lincoln is the lead organisation for this study and will be the data

controller for this study. This means that we are responsible for looking after your information and using it properly.
The university's **Research Participant Privacy Notice** (https://ethics.lincoln.ac.uk/research-privacy-notice/) explains how we will be using information from you in order to undertake this study. If you feel that we have let you down in relation to your information rights then please contact the Information Compliance Team by email on compliance@lincoln.ac.uk or by post at Information Compliance, Secretariat, University of Lincoln, Brayford Pool, Lincoln, LN6 7TS.
You can also make complaints directly to the Information Commissioner's Office (ICO). The ICO is the independent authority upholding information rights for the UK. Their website is ico.org.uk and their telephone helpline number is 0303 123 1113.

Ethics reference: UoL2022_1069

CONSENT TO PARTICIPATE IN RESEARCH

Title of Project: Can resilience be measured as a trait in pet dogs?

Name of Researcher: Dr. Eilidh Mackay

Please read each item carefully and select if you agree (you will not be able to proceed without selecting all).

Please select box

1. I confirm that I have read the information sheet for the above study. I have had the opportunity to consider the information, ask questions and have had these answered satisfactorily.

2. I understand that my participation is voluntary and that I will not be disadvantaged in any way for not taking part.

3. *If I decide to complete the study anonymously*, I understand that it will not be possible to remove any information I have provided, as you will not be able to identify me in any way.

OR

If I decide to provide personal information in the form of an email address, I understand that I can withdraw at any point without giving reason and should I withdraw then the information I

have given up to this point will be deleted/destroyed. However, once the anonymised data set has been created it will not be possible to remove my anonymised data from the analysis.

4. I understand that individuals from the University of Lincoln may look at research data collected during the study, to ensure that the study is conducted appropriately. I give permission for these individuals to have access to my research data.

5. I understand that the information collected about me may be used to support other research in the future, and may be shared anonymously with other researchers.

6. I agree to take part in the above study.

--------------------------------Start of questionnaire---------------------------------

Section 1: Demographic information

1. Which country do you currently live in? (Please select 'Other/Not on list' if the country is not on this list)

(Drop down, select one option)

Anguilla
Antigua and Barbuda
Australia
Bahamas
Barbados
Belize
British Indian Ocean Territory
British Virgin Islands
Canada
Cayman Islands
Dominica
Falkland Islands
Gibraltar
Grenada
Guam
Guernsey
Guyana
Ireland
Isle of Man

Jamaica
Jersey
Montserrat
Nauru
New Zealand
Philippines
Pitcairn
Saint Helena, Ascension and Tristan da Cunha
Saint Kitts and Nevis
Saint Vincent and the Grenadines
Singapore
South Georgia and the South Sandwich Islands
Trinidad and Tobago
Turks and Caicos
United Kingdom
United States
Other/Not on list

2. How old is the dog that you are completing this questionnaire for?

(Multiple choice, select one option)

6 months old or less
6 months – 2 years
2-6 years
6-10 years
10 years +

3. What breed is your dog? (Please select 'Other' if your dog's breed is not stated in this list, please select 'Unknown' if you do not know the breed of your dog, and please select 'Cross-breed' if your dog is a mix between two or more breeds)

(Drop down, select one option)

Affenpinscher
Afghan Hound
Airedale Terrier
Akita
Alaskan Malamute
American Cocker Spaniel
American Water Spaniel
Anatolian Shepherd Dog
Australian Cattle Dog

Australian Kelpie
Australian Shepherd
Australian Silky Terrier
Australian Terrier
Azawakh
Barbet
Basenji
Basset Bleu De Gascogne
Basset Fauve De Bretagne
Basset Griffon Vendeen (Grand)
Basset Griffon Vendeen (Petit)
Basset Hound
Bavarian Mountain Hound
Beagle
Bearded Collie
Beauceron
Bedlington Terrier
Belgian Shepherd (Groenendael)
Belgian Shepherd (Laekenois)
Belgian Shepherd (Malinois)
Belgian Shepherd (Tervueren)
Bergamasco
Bernese Mountain Dog
Bichon Frise
Bloodhound
Bolognese
Border Collie
Border Terrier
Borzoi
Boston Terrier
Bouvier Des Flandres
Boxer
Bracco Italiano
Braque D'Auvergne
Briard
Brittany
Bull Terrier
Bull Terrier (Miniature)
Bulldog
Bullmastiff
Cairn Terrier
Canaan Dog

Canadian Eskimo Dog
Cane Corso (Italian Mastiff)
Catalan Sheepdog
Cavalier King Charles Spaniel
Cesky Terrier
Chesapeake Bay Retriever
Chihuahua (Long Coat)
Chihuahua (Smooth Coat)
Chinese Crested
Chow Chow
Cirneco Dell'Etna
Clumber Spaniel
Cocker Spaniel (English)
Collie (Rough)
Collie (Smooth)
Coonhound (Black & Tan)
Coton De Tulear
Curly Coated Retriever
Dachshund (Long Haired)
Dachshund (Smooth Haired)
Dachshund (Wire Haired)
Dachshund (Miniature Long Haired)
Dachshund (Miniature Smooth Haired)
Dachshund (Miniature Wire Haired)
Dalmatian
Dandie Dinmont Terrier
Deerhound
Doberman
Dogue de Bordeaux
English Pointer
English Setter
English Springer Spaniel
English Toy Terrier (Black & Tan)
Entlebucher Mountain Dog
Estrela Mountain Dog
Eurasier
Field Spaniel
Finnish Lapphund
Finnish Spitz
Flat Coated Retriever
Fox Terrier (Smooth)
Fox Terrier (Wire)

Foxhound
French Bulldog
German Longhaired Pointer
German Shorthaired Pointer
German Wirehaired Pointer
German Pinscher
German Shepherd Dog
German Spitz (Klein)
German Spitz (Mittel)
Glen Of Imaal Terrier
Golden Retriever
Gordon Setter
Grand Bleu De Gascogne
Great Dane
Great Swiss Mountain Dog
Greenland Dog
Greyhound
Griffon Bruxellois
Griffon Fauve De Bretagne
Hamiltonstovare
Harrier Hound
Havanese
Hovawart
Hungarian Kuvasz
Hungarian Puli
Hungarian Pumi
Hungarian Vizsla
Hungarian Wirehaired Vizsla
Ibizan Hound
Irish Red & White Setter
Irish Setter
Irish Terrier
Irish Water Spaniel
Irish Wolfhound
Italian Greyhound
Italian Spinone
Jack Russell Terrier
Japanese Akita Inu
Japanese Chin
Japanese Shiba Inu
Japanese Spitz
Keeshond

Kerry Blue Terrier
King Charles Spaniel
Komondor
Kooikerhondje
Korean Jindo
Korthals Griffon
Labrador Retriever
Lagotto Romagnolo
Lakeland Terrier
Lancashire Heeler
Leonberger
Lhasa Apso
Lowchen
Maltese
Manchester Terrier
Maremma Sheepdog
Mastiff (English)
Mexican Hairless Dog (Xoloitzcuintle) Intermediate
Mexican Hairless Dog (Xoloitzcuintle) Miniature
Mexican Hairless Dog (Xoloitzcuintle) Standard
Miniature Pinscher
Large Munsterlander
Small Munsterlander
Neapolitan Mastiff
Newfoundland
Norfolk Terrier
Norwegian Buhund
Norwegian Elkhound
Norwich Terrier
Nova Scotia Duck Tolling Retriever
Old English Sheepdog
Otterhound
Papillon
Parson Russell Terrier
Pekingese
Pharaoh Hound
Picardy Sheepdog
Polish Lowland Sheepdog
Pomeranian
Poodle (Miniature)
Poodle (Standard)
Poodle (Toy)

Portuguese Podengo
Portuguese Pointer
Portuguese Water Dog
Pug
Pyrenean Mastiff
Pyrenean Mountain Dog
Pyrenean Sheepdog (Long Haired)
Pyrenean Sheepdog (Smooth Faced)
Rhodesian Ridgeback
Rottweiler
Russian Black Terrier
Russian Toy (Russkiy Toy)
Saluki
Samoyed
Schipperke
Schnauzer
Giant Schnauzer
Miniature Schnauzer
Scottish Terrier
Sealyham Terrier
Shar Pei
Shetland Sheepdog
Shih Tzu
Siberian Husky
Skye Terrier
Sloughi
Slovakian Rough Haired Pointer
Soft Coated Wheaten Terrier
Spanish Water Dog
St. Bernard
Staffordshire Bull Terrier
Sussex Spaniel
Swedish Lapphund
Swedish Vallhund
Tibetan Mastiff
Tibetan Spaniel
Tibetan Terrier
Turkish Kangal Dog
Weimaraner
Welsh Corgi (Cardigan)
Welsh Corgi (Pembroke)
Welsh Springer Spaniel

Welsh Terrier
West Highland White Terrier
Whippet
White Swiss Shepherd Dog
Yorkshire Terrier
Crossbreed
Other (Not on list)
Unknown

4. What sex is your dog?
   (Multiple choice, select one option)

Male
Female

5. Is your dog neutered?
   (Multiple choice, select one option)

Yes
No
Don't Know

6. Does your dog have any pre-existing medical conditions?

(Multiple choice, select one option)

Yes
No, not that I am aware of

IF SELECT 'No, not that I am aware of' to above, will be directed straight to question 9. IF SELECT 'Yes' to above, then directed to question 7 and 8 first:

7. Please specify the medical condition(s) below

Box for free text

8. To your knowledge, does this medical condition (or if multiple, does any 1 of them) cause pain? Select 'Yes' even if the pain is occasional or intermittent.

(Multiple choice, select one answer)

Yes

No

9. Have you ever noticed your dog to have an unusual gait or movement?

(Multiple choice, select one answer)

Yes
No, not that I am aware of

10. In your opinion, does your dog ever exhibit any of the following behaviours? Please select all that apply, and please select even if they only do so occasionally.

(Multiple choice, can select multiple answers)

Unfriendly or aggressive behaviour towards people
Unfriendly or aggressive behaviour towards dogs
Separation related behaviour problem
General hyperactivity/overexcitement
Repetitive behaviour including self-mutilation (e.g. compulsive licking/ chewing), tail-chasing or shadow chasing
Fears or phobias e.g. noise reactivity/fear, fear of car travel
Other problem behaviour not listed above
None of the above - no behavioural problems

11. Does your dog currently take any prescription medications?
Please include all forms of medication e.g. oral, injectable (e.g. insulin), eye/ear drops, creams. Include treatments that are administered by the vets also (examples include injectable medications that are given at the vet clinic). Please do NOT include vaccines, worm or flea medications.

(Multiple choice, select one answer)

Yes
No

IF SELECT 'Yes' to above, will be directed to question 12. IF SELECT 'No', will be directed straight to question 13.

12. Please specify the name(s) of the medication(s). If your dog takes multiple medications, please list them all.

Box for free text

13. Does your dog take any supplements or nutraceuticals? e.g. joint, digestive, skin or anxiety supplements – anything that is purchased over the counter WITHOUT requiring veterinary prescription. Do NOT include worming or flea treatment.

(Multiple choice, select one answer)

Yes
No
IF SELECT 'Yes' to above, will be directed to question 14. Otherwise, will be directed straight to question 15.

14. Please specify the name(s) of the product(s). If they take multiple, please include them all.

Box for free text

15. Do you use any of the following pheromone products for your dog? Please select all that apply.

(Multiple choice, can select multiple answers)

Adaptil® On-The-Go Collar for Dogs
Adaptil® Calming Spray for Dogs
Adaptil® Home Diffuser for Dogs
I don't use any of the above

Section 2: An understanding of how your dog copes with stress

Now we would like to get an understanding of how your dog copes with stress.

For each of the statements below, please select the response which most accurately describes how you feel your dog behaves in this situation, **at present**. We know this can be hard, but try to think how they would behave *in general* in the stated scenario.

If your dog has never experienced the situation, and you don't feel that you can accurately predict their behaviour in this context, please select the "N/A or Don't Know" option.

	**Strongly Agree**	**Mainly Agree**	**Partly Agree, Partly Disagree**	**Mainly Disagree**	**Strongly Disagree**	**N/A or Don't Know**

My dog takes a long time to recover from setbacks in life e.g. loss of a companion, knock in confidence	○	○	○	○	○	○
My dog bounces back quickly from illness or injury	○	○	○	○	○	○
If something upsets my dog, they tend to get over it quickly	○	○	○	○	○	○
If something frightened my dog, he/she would be nervous to return to that place for a long time afterwards	○	○	○	○	○	○
If my dog were to have a bad experience with another individual (dog or person), he/she would forget about it quickly and not hold onto it	○	○	○	○	○	○
If something were to startle or frighten my dog, he/she would remain on edge for some time afterwards	○	○	○	○	○	○

My dog always tries his/her hardest even when the task is difficult	○	○	○	○	○	○
My dog does not get upset easily	○	○	○	○	○	○
My dog generally takes stressful situations in their stride	○	○	○	○	○	○
My dog is not easily discouraged if he/she does not succeed in something the first time e.g. learning a new trick, trying to solve a puzzle	○	○	○	○	○	○
My dog will sometimes seem out of sorts for no apparent reason	○	○	○	○	○	○
If another dog had a negative reaction to something, my dog is likely to become upset too	○	○	○	○	○	○
My dog does not cope well with change e.g. changes in work hours, house move	○	○	○	○	○	○
My dog enjoys anything that is new and unusual e.g.						

objects, animals or anything they have not seen before	○	○	○	○	○	○
I would regard my dog to be very adaptable i.e. able to fit into any situation	○	○	○	○	○	○
I would regard my dog to be very curious/open to new experiences e.g. enjoys exploring new environments	○	○	○	○	○	○
My dog enjoys challenges e.g. learning new tricks, finding hidden items, solving a difficult task	○	○	○	○	○	○
If something unexpected were to happen and my life circumstances were to change, I know my dog could cope	○	○	○	○	○	○
I believe my dog to be a resilient individual i.e. able to cope with, 'bounce back' from and/or adapt to adversity or change	○	○	○	○	○	○

My dog generally gets along well with other dogs	○	○	○	○	○	○
My dog generally gets along well with other people	○	○	○	○	○	○
My dog is easily frustrated	○	○	○	○	○	○
I believe my dog to be self-confident	○	○	○	○	○	○
I believe my dog to be an anxious individual	○	○	○	○	○	○
I would describe my dog as strong-willed/determined	○	○	○	○	○	○
I would describe my dog as a calm, even-tempered individual	○	○	○	○	○	○

-------------------------------End of questionnaire items-------------------------------

If you are happy to be contacted to complete this questionnaire again for the ***same dog*** in 6 weeks' time, please leave your email address below (please refer to participant information sheet regarding handling of personal information). Otherwise, please leave blank.

Box for free text

IF above is NOT left empty, then will be directed to the final question:

Would you like the results of this research to be sent to the email address provided? (If left blank, 'No' will be default)

(Multiple choice, select one answer)

Yes
No

S3: Basic demographic information relating to initial survey

Categorisation of Breeds of respondents

Breed Category	Count	Percentage in dataset
Border Collie	102	9.4
Labrador Retriever	78	7.2
Cocker Spaniel (English)	58	5.4
German Shepherd Dog	28	2.6
English Springer Spaniel	28	2.6
Golden Retriever	21	1.9
Jack Russell Terrier	20	1.8
Greyhound	17	1.6
Staffordshire Bull Terrier	16	1.5
Australian Shepherd	14	1.3
Miniature Schnauzer	13	1.2
Cross-breed	322	29.7
'Other pure-breed'	272	25.1

'Other pure-breed' incorporated all other pure breeds that accounted for <1% of entire dataset, for the purposes of statistical analyses.

Age distribution of dogs

Age category	Number (n)	Percentage (%)
6 months old or less	5	0.5
6 months – 2 years	135	12.5
2-6 years	424	39.1
6-10 years	273	25.2
10 years +	224	20.7
Unanswered	23	2.1
**TOTAL**	1084	100.0

S4: Initial metrics on item quality

Percentage score agreement for each item between the first and second completion of the questionnaire.

Item	Percentage Agreement (%)
Item 1	50.0
Item 2	56.3
Item 3	48.6
Item 4	45.5
Item 5	37.9
Item 6	51.4
Item 7	54.4
Item 8	43.7
Item 9	50.2
Item 10	49.8
Item 11	47.3
Item 12	41.1
Item 13	39.9
Item 14	41.5
Item 15	47.2
Item 16	57.4
Item 17	57.9
Item 18	48.1
Item 19	55.4

6 week intra-rater reliability analysis

(* = items removed based on Spearman's correlation <0.2 and/or Wilcoxon p<0.05).

Item number	Spearman's rho	p (Bonferroni-adjusted)	Wilcoxon p (Bonferroni-adjusted)
1	0.583	<0.001	**0.002***
2	0.463	<0.001	1.000
3	0.648	<0.001	**0.012***
4	0.591	<0.001	0.573
5	0.512	<0.001	1.000
6	0.688	<0.001	1.000
7	0.629	<0.001	1.000
8	0.624	<0.001	1.000
9	0.716	<0.001	0.631
10	0.592	<0.001	0.251
11	0.547	<0.001	0.144
12	0.586	<0.001	1.000

13	0.598	<0.001	**0.033***
14	0.540	<0.001	1.000
15	0.679	<0.001	1.000
16	0.625	<0.001	1.000
17	0.626	<0.001	1.000
18	0.550	<0.001	0.577
19	0.715	<0.001	1.000

S5: Inter-item Correlations and Principal Component Analysis (PCA)

Correlation Matrix of Items

Correlation matrix

	Item 2	Item 4	Item 5	Item 6	Item 7	Item 8	Item 9	Item 10	Item 11	Item 12	Item 14	Item 15	Item 16	Item 17	Item 18	Item 19
Item 2	1.00	0.28	0.22	0.27	0.27	0.28	0.30	0.25	0.27	0.17	0.27	0.36	0.39	0.27	0.33	0.41
Item 4	0.28	1.00	0.44	0.63	0.16	0.47	0.53	0.17	0.42	0.38	0.38	0.42	0.40	0.16	0.38	0.49
Item 5	0.22	0.44	1.00	0.50	0.15	0.52	0.51	0.14	0.28	0.37	0.40	0.49	0.35	0.12	0.40	0.51
Item 6	0.27	0.63	0.50	1.00	0.18	0.59	0.61	0.17	0.50	0.44	0.44	0.53	0.46	0.15	0.44	0.59
Item 7	0.27	0.16	0.15	0.18	1.00	0.21	0.28	0.67	0.20	0.08	0.31	0.28	0.35	0.63	0.30	0.32
Item 8	0.28	0.47	0.52	0.59	0.21	1.00	0.64	0.21	0.41	0.43	0.45	0.61	0.42	0.12	0.51	0.62
Item 9	0.30	0.53	0.51	0.61	0.28	0.64	1.00	0.25	0.42	0.36	0.52	0.67	0.51	0.21	0.61	0.69
Item 10	0.25	0.17	0.14	0.17	0.67	0.21	0.25	1.00	0.24	0.07	0.31	0.24	0.31	0.60	0.28	0.32
Item 11	0.27	0.42	0.28	0.50	0.20	0.41	0.42	0.24	1.00	0.28	0.29	0.33	0.27	0.18	0.36	0.41
Item 12	0.17	0.38	0.37	0.44	0.08	0.43	0.36	0.07	0.28	1.00	0.27	0.33	0.28	0.08	0.24	0.36
Item 14	0.27	0.38	0.40	0.44	0.31	0.45	0.52	0.31	0.29	0.27	1.00	0.54	0.59	0.23	0.44	0.51
Item 15	0.36	0.42	0.49	0.53	0.28	0.61	0.67	0.24	0.33	0.33	0.54	1.00	0.56	0.16	0.59	0.71
Item 16	0.39	0.40	0.35	0.46	0.35	0.42	0.51	0.31	0.27	0.28	0.59	0.56	1.00	0.34	0.44	0.53

Item 17	0.27	0.16	0.12	0.15	0.63	0.12	0.21	0.60	0.18	0.08	0.23	0.16	0.34	1.00	0.21	0.18
Item 18	0.33	0.38	0.40	0.44	0.30	0.51	0.61	0.28	0.36	0.24	0.44	0.59	0.44	0.21	1.00	0.61
Item 19	0.41	0.49	0.51	0.59	0.32	0.62	0.69	0.32	0.41	0.36	0.51	0.71	0.53	0.18	0.61	1.00

Extraction of Principal Components

Total Variance Explained

	Initial Eigenvalues			Rotation Sums of Squared Loadings		
Component	Total	% of Variance	Cumulative %	Total	% of Variance	Cumulative %
1	6.818*	42.613	42.613	6.02	38	38
2	2.017*	12.609	55.222	2.82	18	55
3	0.976	6.100	61.322			
4	0.820	5.126	66.448			
5	0.771	4.820	71.268			
6	0.682	4.260	75.528			
7	0.597	3.734	79.262			
8	0.527	3.294	82.556			
9	0.457	2.856	85.412			
10	0.429	2.680	88.092			
11	0.377	2.358	90.450			
12	0.342	2.135	92.585			
13	0.326	2.036	94.621			
14	0.313	1.954	96.575			
15	0.289	1.804	98.379			
16	0.259	1.621	100.000			

Extraction Method: Principal Component Analysis.

**Components to be retained with Eigen value >1*

Scree plot of components identified in the PCA.

Loadings of items onto Principal Components

Extraction Method: Principal Component Analysis.

Rotation Method: Varimax with Kaiser Normalization.

Rotated Component Matrix[a]

	Component	
	1	2
Item 2*	0.38	0.37
Item 4	**0.70**	0.07
Item 5	**0.69**	0.03
Item 6	**0.80**	0.07
Item 7	0.13	**0.86**
Item 8	**0.79**	0.09
Item 9	**0.80**	0.20
Item 10	0.12	**0.84**
Item 11	**0.54**	0.17
Item 12	**0.57**	-0.06
Item 14	**0.61**	0.32
Item 15	**0.77**	0.21
Item 16*	**0.59**	**0.41**
Item 17	0.05	**0.83**
Item 18	**0.65**	0.28
Item 19 (GeneralmValid-ity Item)	**0.79**	0.25

*Items in **BOLD** text indicate items loading >0.4*

** = Items removed based on insufficient loading (<0.4) onto either component (Item 2), or cross-loading onto >1 component (Item 16):*

**Item number**	**Item**
**Item 2**	My dog bounces back quickly from illness or injury
**Item 16**	I would regard my dog to be curious/open to new experiences e.g. enjoys exploring new environments

S6: Metrics relating to comparison of 'complete' and 'incomplete' data sets for The Canine Adaptability and Resilience Scale (CARS)

Wilcoxon rank sum tests on 'complete' and 'incomplete' data sets

i.e., Comparison of dataset used for PCA (i.e. only 'complete' responses) with the dataset that was removed before PCA analysis (responses with missing entries) to determine if they show any significant differences. No significant differences found

Component	W	p-value	Bonferroni-adjusted p value
**PC1 Score**	438466	0.4842	1.00
**PC2 Score**	444049	0.2332	1.00
**Item 4**	426090	0.9069	1.00
**Item 5**	422559	0.8757	1.00
**Item 6**	438118	0.4903	1.00
**Item 7**	433749	0.3516	1.00
**Item 8**	430865	0.9074	1.00
**Item 9**	436214	0.4184	1.00
**Item 10**	435681	0.4159	1.00
**Item 11**	430631	0.7246	1.00
**Item 12**	407588	0.8926	1.00
**Item 14**	437790	0.4180	1.00
**Item 15**	438409	0.4282	1.00
**Item 17**	432206	0.6629	1.00
**Item 18**	433787	0.3533	1.00
**Item 19**	435421	0.5223	1.00

S7: The Canine Adaptability Resilience Scale (CARS)

See text for details on scoring. (R) = reverse scored items

	**Strongly Agree**	**Mainly Agree**	**Partly Agree, Partly Disagree**	**Mainly Disagree**	**Strongly Disagree**	**N/A or Don' t Know**
If something frightened my dog, he/she would be nervous to return to that location for a long time afterwards **(R)**						
If my dog were to have a bad experience with another individual (dog or person), he/she would forget about it quickly and not hold onto it						
If something were to startle or frighten my dog, he/she would remain on edge for some time afterwards **(R)**						
My dog always tries his/her hardest even when the task is difficult						
My dog does not get upset easily						
My dog generally takes stressful situations in their stride						
My dog will persevere even when they do not succeed in something straight away e.g. when learning a new trick, when trying to solve a puzzle etc.						
My dog will sometimes seem out of sorts for no apparent reason **(R)**						
If another dog has a negative reaction to something, my dog is likely to become upset too **(R)**						
My dog enjoys anything that is new or unusual e.g. objects, animals or anything they have not seen before						
I would regard my dog to be very adaptable i.e. able to fit into any situation						
My dog enjoys challenges e.g. learning new tricks, finding hidden items, solving a difficult task						
If something unexpected were to happen and my life circumstances were to change, I know my dog could cope						
I believe my dog to be a resilient individual i.e. able to cope with, 'bounce back' from and/or adapt to adversity or change						

S8: Metrics relating to final questionnaire: The Canine Adaptability and Resilience Scale (CARS)

Internal consistency

Calculated for 'complete' datasets only (n = 794)

	**Cronbach's alpha score**
**Overall**	0.896
**After removal of PC1**	0.837
**After removal of PC2**	0.907

Demographic variables explored in relation to Principal Component Scores

**Fixed Factor**	**Groups**
**Country**	United Kingdom
	United States
	Other countries
**Age**	6 months old or less
	6 months - 2 years
	2 - 6 years
	6 - 10 years
	10 years +
**Breed**	Border Collie
	Labrador Retriever
	Cocker Spaniel (English)
	German Shepherd Dog
	English Springer Spaniel
	Golden Retriever
	Jack Russell Terrier
	Greyhound
	Staffordshire Bull Terrier
	Australian Shepherd
	Miniature Schnau-zer
	Cross-Breed
	Other Pure-Breed
**Sex**	Male

	Female
**Neuter Status**	Neutered
	Unneutered
**Medical Condition**	Present
	Absent
**Takes prescription medications**	Yes
	No
**Takes supplements or nutraceuticals**	Yes
	No
**Uses pheromone products (diffuser, spray and/or collar)**	Yes
	No
**Problem behaviour: Aggressive towards people**	Yes
	No
**Problem behaviour: Aggressive towards dogs**	Yes
	No
**Problem behaviour: Separation related behaviour problem**	Yes
	No
**Problem behaviour: General hyperactivity/overexcitement**	Yes
	No
**Problem behaviour: Repetitive behaviour**	Yes
	No
**Problem behaviour: Fears or phobias**	Yes
	No
**Problem behaviour: Other**	Yes
	No

Range, mean, standard deviation, median and skewness of the two components making up Canine Adaptability and Resilience Scale.

**Component**	**Total range (min-max score in dataset)**	**Mean**	**Standard Deviation (SD)**	**Median**	**Skewness**
PC1 Adaptability/ behavioural flexibility	0.22-1.00	0.70	0.17	0.71	-0.40
PC2 Perseverance	0.20-1.00	0.80	0.18	0.80	-0.96
